# Integrating climate model projections into environmental risk assessment: A probabilistic modeling approach

**DOI:** 10.1002/ieam.4879

**Published:** 2024-01-10

**Authors:** S. Jannicke Moe, Kevin V. Brix, Wayne G. Landis, Jenny L. Stauber, John F. Carriger, John D. Hader, Taro Kunimitsu, Sophie Mentzel, Rory Nathan, Pamela D. Noyes, Rik Oldenkamp, Jason R. Rohr, Paul J. van den Brink, Julie Verheyen, Rasmus E. Benestad

**Affiliations:** 1 Norwegian Institute for Water Research (NIVA), Oslo, Norway; 2 EcoTox LLC, Miami, Florida, USA; 3 RSMAES, University of Miami, Miami, Florida, USA; 4 College of the Environment, Western Washington University, Bellingham, Washington, USA; 5 CSIRO Environment, Lucas Heights, Sydney, NSW, Australia; 6 La Trobe University, Wodonga, Victoria, Australia; 7 Center for Environmental Solutions and Emergency Response, Office of Research and Development, USEPA, Land Remediation and Technology Division, Cincinnati, Ohio, USA; 8 Department of Environmental Science, Stockholm University, Stockholm, Sweden; 9 CICERO Center for International Climate Research, Oslo, Norway; 10 Department of Infrastructure Engineering, University of Melbourne, Melbourne, Victoria, Australia; 11 Center for Public Health and Environmental Assessment, Office of Research and Development, USEPA, Integrated Climate Sciences Division, Washington, DC, USA; 12 Chemistry for Environment and Health, Vrije Universiteit Amsterdam, Amsterdam, The Netherlands; 13 Department of Biological Sciences, University of Notre Dame, Notre Dame, Indiana, USA; 14 Aquatic Ecology and Water Quality Management Group, Wageningen University, Wageningen, The Netherlands; 15 Laboratory of Evolutionary Stress Ecology and Ecotoxicology, KU Leuven, Belgium; 16 The Norwegian Meteorological Institute, Oslo, Norway

**Keywords:** Bayesian network, Climate information, Downscaling, Exposure model, Probabilistic risk assessment

## Abstract

The Society of Environmental Toxicology and Chemistry (SETAC) convened a Pellston workshop in 2022 to examine how information on climate change could be better incorporated into the ecological risk assessment (ERA) process for chemicals as well as other environmental stressors. A major impetus for this workshop is that climate change can affect components of ecological risks in multiple direct and indirect ways, including the use patterns and environmental exposure pathways of chemical stressors such as pesticides, the toxicity of chemicals in receiving environments, and the vulnerability of species of concern related to habitat quality and use. This article explores a modeling approach for integrating climate model projections into the assessment of near- and long-term ecological risks, developed in collaboration with climate scientists. State-of-the-art global climate modeling and downscaling techniques may enable climate projections at scales appropriate for the study area. It is, however, also important to realize the limitations of individual global climate models and make use of climate model ensembles represented by statistical properties. Here, we present a probabilistic modeling approach aiming to combine projected climatic variables as well as the associated uncertainties from climate model ensembles in conjunction with ERA pathways. We draw upon three examples of ERA that utilized Bayesian networks for this purpose and that also represent methodological advancements for better prediction of future risks to ecosystems. We envision that the modeling approach developed from this international collaboration will contribute to better assessment and management of risks from chemical stressors in a changing climate.

## INTRODUCTION

Current and projected global climate change will affect the physical environment and biological diversity in both terrestrial and aquatic ecosystems. This includes combined and interactive effects of climate change with anthropogenic changes in chemical, physical, and biological stressors. Improving the methodologies to assess the risk of chemical stressors in the context of climate scenarios and climate model projections will provide a better foundation for the future management of chemicals (Cains et al., 2023), and more generally for environmental management adapted to climate change.

The 6th Assessment Report of the Intergovernmental Panel on Climate Change (IPCC AR6) states with high confidence that ecosystem damage by pollutants, together with habitat fragmentation and unsustainable use of natural resources, will increase ecosystem vulnerability to climate change globally, even within protected areas (SPM.B.2.2) (IPCC, 2022). Moreover, numerous potential interactions between climate change and chemical stressors have been highlighted, for example, by the European Environment—State and Outlook 2020 Report (European Environment Agency [EEA], 2019): accumulated chemicals in soil sediment and ice will be increasingly remobilized by storms, ice melting, or flooding, due to the increasing frequency and magnitude of such events. Nevertheless, the potential influences of climate change have not yet been systematically incorporated into frameworks for environmental assessments of chemical and other stressors (e.g., EEA, 2018).

**FIGURE 1:**
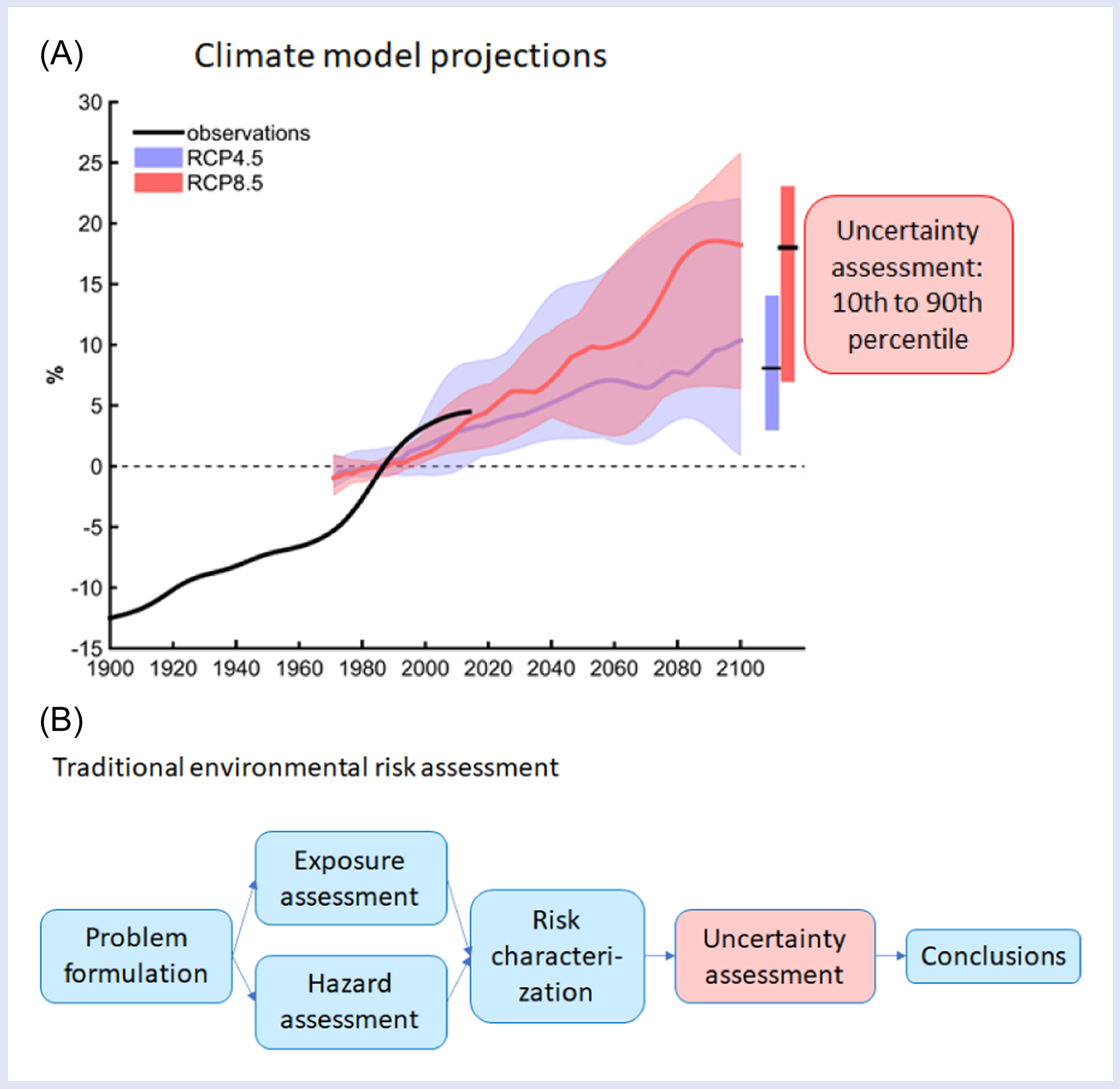
Examples of representation of uncertainty assessment: in climate model projections (A) and in traditional ERA frameworks (B). The graph (A) represents annual precipitation over Norway as percentage deviation (%) from the period 1971 to 2000, modified after The Norwegian Centre for Climate Services (Hanssen-Bauer et al., 2017). The flow chart (B) is redrawn after the SETAC (2018) Technical Issue Paper on environmental risk assessment. ERA, environmental risk assessment; SETAC, Society of Environmental Toxicology and Chemistry

A call for research on global climate change and environmental contaminants by the Society of Environmental Toxicology and Chemistry (SETAC) (Wenning et al., 2010) resulted in a compilation of current knowledge on climate change impacts on chemical exposure and vulnerability of organisms, populations, communities, and ecosystems (Hooper et al., 2013; Moe et al., 2013; Stahl, Jr et al., 2013), and identification of seven principles for integrating climate change into environmental risk assessment (ERA) of chemical stressors (Landis et al., 2013). However, barriers to incorporating climate change into traditional ERA frameworks and methodology were also recognized (Landis et al., 2014). For example, there are large uncertainties associated with projections of climate variables, even within any given emission scenario (Figure 1A). This is difficult to handle explicitly and transparently within the traditional practices of ERA, where uncertainty sources tend to be merged into single quantities such as assessment factors (Figure 1B).

To bridge this gap, a SETAC Pellston workshop^®^ was organized in June 2022 to stimulate collaboration between climate modelers and environmental risk experts (Stahl Jr. et al., 2023). The participants identified and discussed numerous examples of ecosystems where environmental risks of chemicals are known to be influenced by climate change. A more detailed description of environmental processes as well as societal and institutional processes relevant for chemical risk management is given by Cains et al. (2023). Three of these examples were selected as case studies (Table 1) for the purpose of developing and evaluating a quantitative modeling approach for integrating climate model projections into ERA.

Preliminary key messages from this exercise (Moe et al., 2022) stated that better integration of the two disciplines will benefit from (as discussed in the section “Climate information”) the following: projections from ensembles of global climate models (GCMs) (Lee et al., 2021), regional downscaling of future climate projections to suitable spatial scales by dynamical and empirical–statistical downscaling techniques (Doblas-Reyes et al., 2021; Erlandsen et al., 2020), temporal aggregation of climate projections in the form of probability distribution functions represented by statistical parameters (Benestad, Parding, Erlandsen, et al., 2019) and incorporation of the resulting probability distributions, henceforth referred to as “climate information,” into environmental exposure and ecological effect assessments by probabilistic modeling techniques.

A principle in climate modeling is the need for large ensembles of GCMs to capture the uncertainty associated with individual models (Deser et al., 2012; Lee et al., 2021). However, the use of GCMs for use in local-scale assessment and decision-making poses many challenges (Doblas-Reyes et al., 2021; Ranasinghe et al., 2021; Wilby & Dessai, 2010), some of which will be addressed in this article. We use the term “climate information” (e.g., Nilsen et al., 2022) to refer to quantitative information derived from climate model projections, which is robust to model assumptions, representative of the most recent knowledge available, and relevant for the specific case study. Such interdisciplinary collaboration is needed to identify and quantify the most important elements of the physical environment (e.g., air, water, and soil temperature; precipitation; wind; evaporation; air pressure) influencing ecosystem processes and to obtain both statistical robustness (large enough sample) and relevance for the ecosystem (spatial scale) (John et al., 2021). Moreover, probabilistic approaches are needed to combine the propagation of uncertainty in climate projections, weather stochasticity, and model parameters into a probabilistic characterization of risk (Maertens et al., 2022).

**FIGURE 2:**
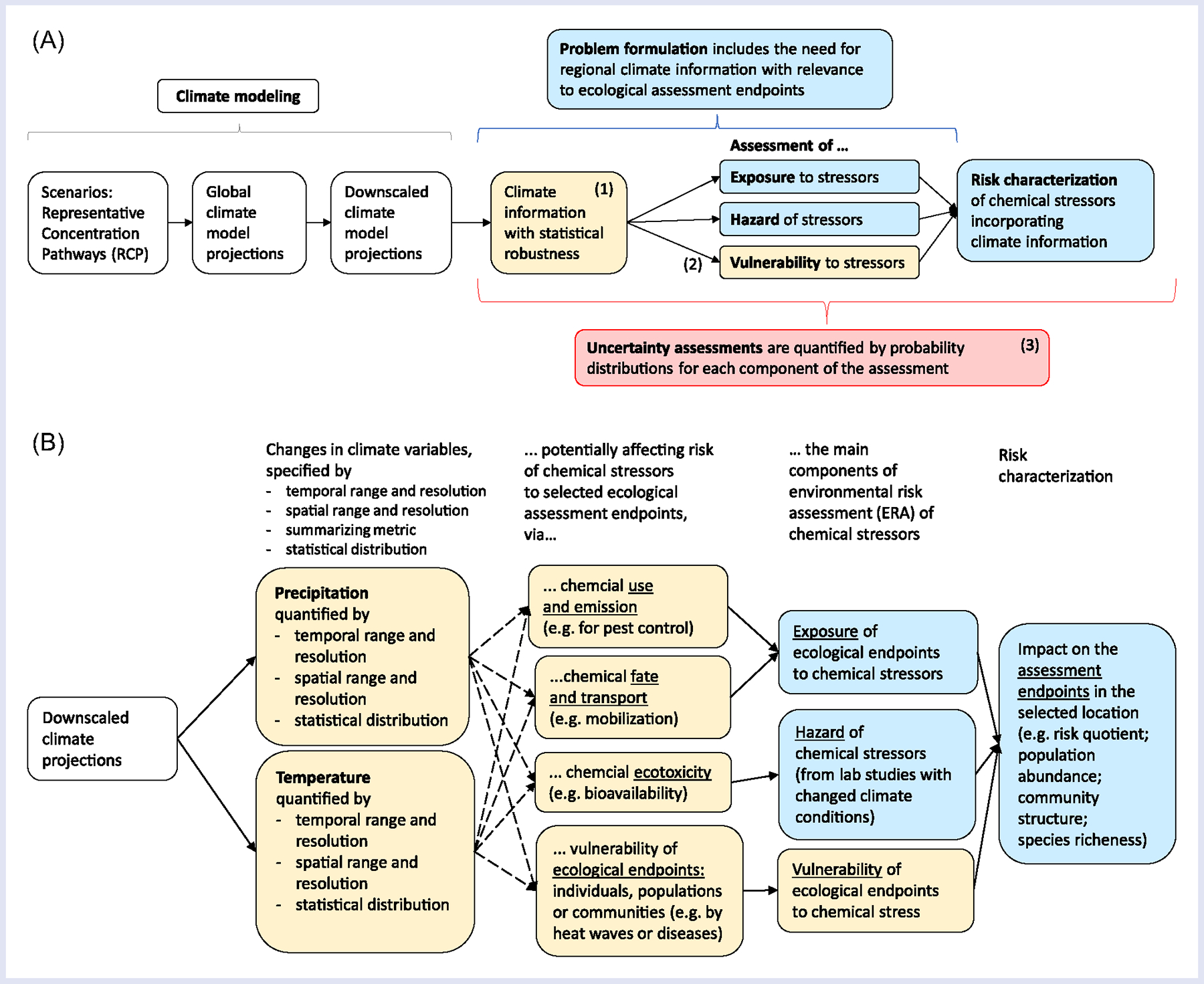
Proposed modeling approach for integration of climate model projections into environmental risk assessment (ERA) of chemical stressors. (A) Compared to traditional ERA (Figure 1B), the main novel aspects are as follows: (1) derivation of robust and relevant climate information; (2) assessment of climate-induced vulnerability to chemical stress; and (3) use of probabilistic and (preferably) causal modeling methodology for integrating the components of the risk characterization. (B) Examples of climate information and potential influence on the main components of ERA

Overall, the workshop confirmed the need for collaboration between climate modelers and environmental scientists to coproduce knowledge on climate change (cf. Chambers et al., 2021). The objective of this article is to propose, describe, and evaluate a modeling approach for integrating climate model projections with quantitative approaches to ERA of chemical stressors. In this article, we use the terms “chemical stressor” or “contaminants” to include both hazardous substances and nutrients. For this purpose, we have considered state-of-the-art methodologies within both climate modeling and environmental risk modeling to find common ground and optimize possibilities for connecting information from the two scientific fields (Figure 2). In brief, the three pillars representing main novel aspects of the proposed modeling approach are as follows:

1. Climate information: derivation and use of relevant and robust climate information represented by statistical properties of climate model projections.
2. Climate-induced vulnerability: consideration of how climate change can modify the sensitivity of individuals to chemicals in a natural ecosystem, included here as a third component of environmental risk characterization, and
3. Probabilistic modeling: use of Bayesian networks (BNs) as a probabilistic and potentially causal modeling methodology for integrating climate information represented by statistical properties into the risk components.

**FIGURE 3:**
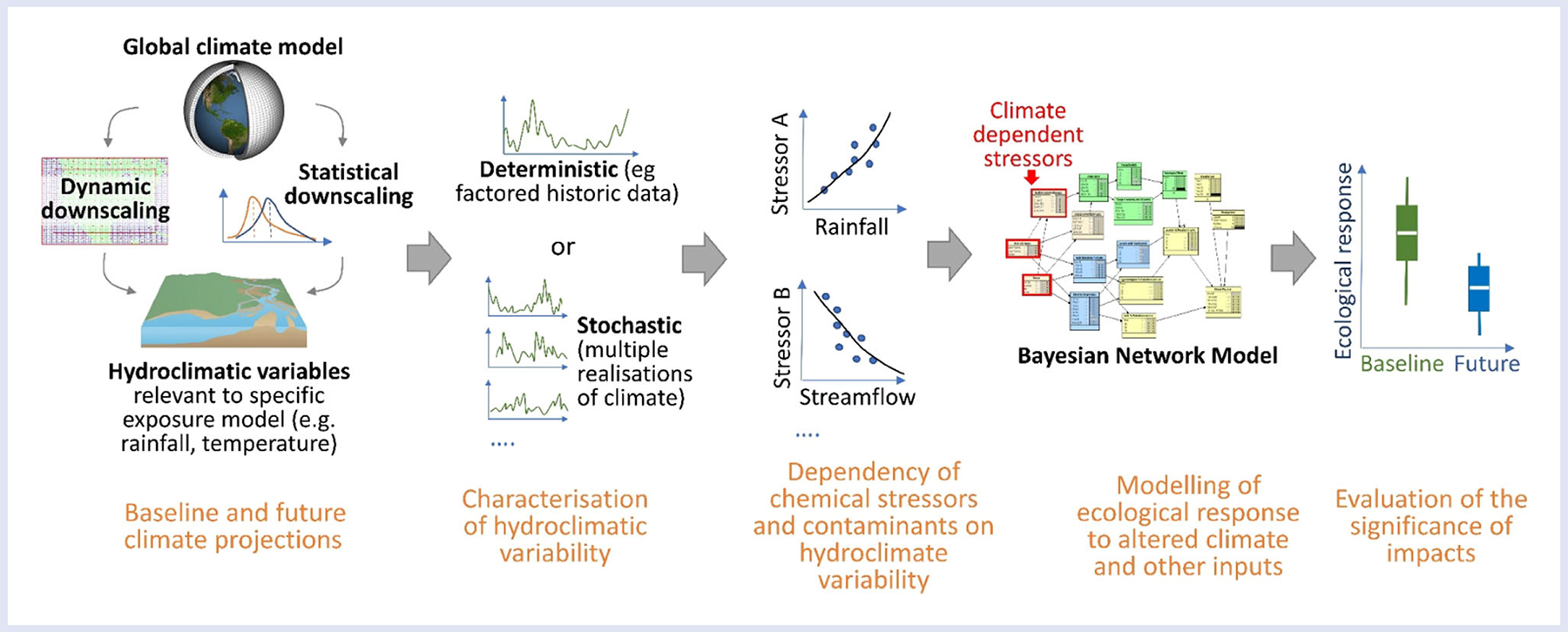
Schematic illustration and example of work flow for integrating climate model projections with risk characterization, as exemplified by the three case studies

Initial applications of this modeling approach are demonstrated in three case studies resulting from the workshop (Table 1), which are described in more detail in three respective papers (Landis et al., 2023; Mentzel et al., 2023; Oldenkamp et al., 2023) and summarized in Tables 2 and 3. The three case studies are based on discussions and analyses carried out during the months following the workshop (Stahl Jr. et al., 2023). The stages of model development range from de novo and preliminary model construction that does not yet fully integrate the various assessment endpoints (Mentzel et al., 2023) to a post-hoc incorporation of climate change into an established ERA model (Landis et al., 2023). The main steps of deriving and processing future climate information and integrating it with a complete risk assessment are outlined schematically in Figure 3. Rather than completing all the steps outlined in Figure 3, the purpose of the resulting case study papers is to demonstrate some of the steps needed to incorporate climate projections into a probabilistic ERA, to give examples of relevant information and assumptions, and to highlight opportunities and limitations of this approach.

## MODELING APPROACH AND BACKGROUND INFORMATION

### Concepts and principles

An overview of relevant concepts related to climate modeling is presented in Table 4. Scientific communities usually define “risk” in terms of both probability and consequence, but in practice, these two elements are often combined into a single quantity or score (e.g., as the product), even in meteorological communities (Palmer & Richardson, 2014). In the guidance for IPCC authors (Reisinger et al., 2020), the core definition of risk to human and ecological systems is “the potential for adverse consequences,” which includes uncertainty as the potential for an outcome. According to this guidance, “This uncertainty does not necessarily have to be quantified, but authors need to provide some sense of the nature and degree of uncertainty to allow a meaningful risk assessment and risk management responses to be undertaken.” The probabilistic methodology described in the section “Integration of climate and risk components” furthermore provides a tool for incorporating uncertainty, provided that the uncertainty can be quantified by probabilities.

The IPCC has described risk in terms of the three components: exposure, hazard, and vulnerability (Reisinger et al., 2020) “In the context of climate change impacts, risks result from dynamic interactions between climate-related hazards with the exposure and vulnerability of the affected human or ecological system to the hazards. Hazards, exposure and vulnerability may each be subject to uncertainty in terms of magnitude and likelihood of occurrence, and each may change over time and space due to socioeconomic changes and human decision-making.” Here, we will consider vulnerability as a modifying factor of chemical risk in addition to exposure and hazard, inspired by (but not identical to) IPCC’s definition, and describe the potential influence of climate change on all three components (Figure 2).

In this context, hazard can be interpreted as the response of organisms exposed to chemicals under standard or modified laboratory test conditions (usually representing the individual level), while vulnerability can be interpreted as the response of the organisms to climate change within a natural ecosystem, affected by physical disturbance, habitat quality, species interactions, and so forth (community level). In the following subsections, we will describe examples of how climate change can influence each of these components, with reference to the case studies as well as to other recent research methods applied for this purpose.

The first SETAC Pellston workshop on global climate change resulted in a list of seven principles for guiding the decision on when and how to incorporate climate change information into ERA (Landis et al., 2013).

1. Consider the importance of global climate change-related factors in the ERA process and subsequent management decisions.
2. Assessment endpoints should be expressed as ecosystem services.
3. Responses of endpoints can be positive or negative.
4. The ERA process requires a multiple-stressor approach, and responses may be nonlinear.
5. Develop conceptual cause–effect diagrams that consider relevant management decisions as well as appropriate spatial and temporal scales to allow consideration of both direct and indirect effects of climate change.
6. Determine the major drivers of uncertainty, estimating and bounding stochastic uncertainty spatially and temporally, and continue the process as management activities are implemented.
7. Plan for adaptive management to account for changing environmental conditions and consequent changes to endpoints.

The authors furthermore suggested that the nature of the interaction of climate change with chemicals and other stressors requires approaches that depict the probabilistic nature of the system, and that analyses of such systems may benefit from the use of BNs. Here, while building upon these seven principles, we focus on the probabilistic modeling methodology for chemical risk and other specific considerations for incorporating climate model projections. For this purpose, in the following, we describe the traditional components of chemical risk (exposure and hazard) as well as vulnerability (as defined in the Introduction) and present examples of how climate change can influence each component (summarized in Table 3; examples in Figure 2B).

### Components of chemical risk and influence of climate change

#### Climate and chemical exposure.

**FIGURE 4:**
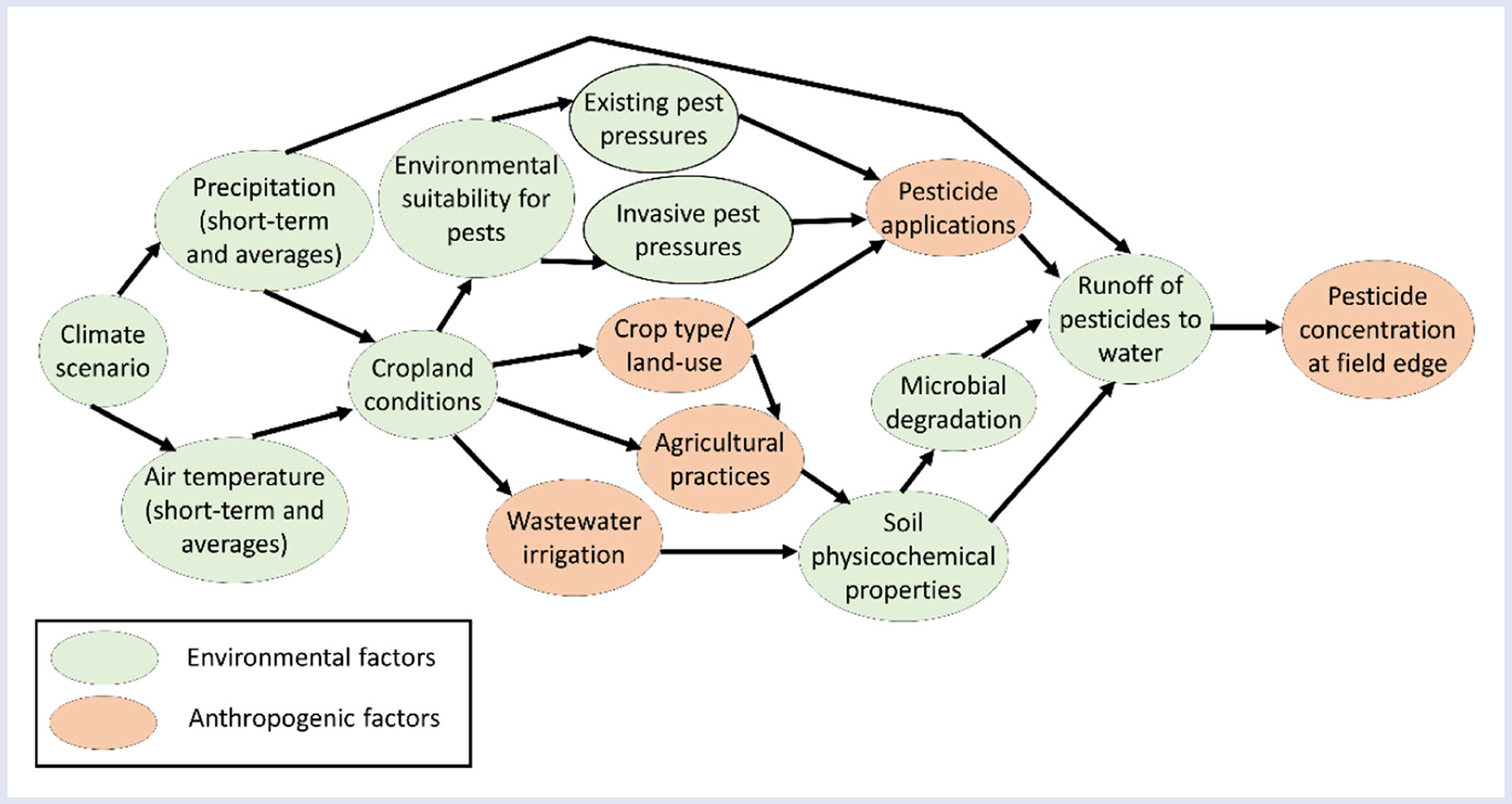
Example of potential causal impacts from climate change on exposure to pesticides in an aquatic environment considering environmental factors (i.e., direct effects) and anthropogenic factors (i.e., indirect effects). Based on information in Hader et al. (2022)

The IPCC recognizes that risks can arise from direct impacts of climate change, as well as indirectly via human responses to climate change (IPCC, 2022; Reisinger et al., 2020). In this vein, we emphasize both the direct environmental and indirect anthropogenic factors that can impact chemical exposure in the context of climate change. Hader et al. (2022) reviewed how global change (i.e., climate change, society’s responses to these changes, and other large-scale societal changes) may impact the emission, persistence, fate, and transport of chemicals in agricultural settings in Europe. An example of the interlinkages between direct climate change and indirect anthropogenic driving forces on pesticide exposure in an aquatic environment influenced by agriculture is outlined in Figure 4, based on some of the findings of the review (Hader et al., 2022). Changes in environmental conditions can affect both how suitable an environment may be for agricultural pest species as well as the suitability of the land for different crops. In turn, increases in pest pressures may result in increased pesticide usage. Changes in field conditions may impact the types of crops grown and other agricultural practices, which can impact the soil’s physicochemical properties, which can in turn impact the capacity for microbial degradation of pesticides in the environment. Changes in precipitation patterns can then directly affect how much pesticide runs off from the agricultural soil into an adjacent water body. Likewise, the emissions, fate, and transport of other chemicals could be impacted by climate change and attendant human responses. For example, increased intensity and frequency of droughts could increase the use of wastewater for irrigation in some regions (e.g., southern Europe), resulting in new exposure pathways of pharmaceuticals and other consumer product residues present after the water treatment process (Hader et al., 2022).

A key component of understanding chemical exposure under climate change conditions is understanding how chemical emissions may change as society adapts to changed environmental conditions. For example, in the context of agriculture and pesticide emissions, two different approaches have been adopted across studies. The first utilizes regression modeling between pesticide application amounts and weather or climate variables, and then uses projections of future climate variables to project changes in pesticide application (e.g., Chiu et al., 2017; Kattwinkel et al., 2011). The second approach relates weather or climate variables to pest growth, physiology, and*/*or behavior; uses climate projections to predict how these pests may respond to these environmental changes; and pairs this with available information on chemical treatment practices (e.g., Gagnon et al., 2016; Steffens et al., 2015). Further details on these two methods and how they can be used to help incorporate climate change into a probabilistic ERA are described in Oldenkamp et al. (2023).

Several other studies have focused on other aspects of climate change and influence on chemical exposure, for example, on the duration of pesticide exposure (Rohr et al., 2011), on chemical fate and bioaccumulation (Gouin et al., 2013), and on factors related to resource damage assessment and restoration (Rohr et al., 2013). The multifaceted nature of the impact of climate change on chemical emissions, fate, and transport, and ultimately environmental exposure, and the attendant uncertainties associated with each facet highlight the need for a probabilistic, causal modeling approach.

#### Climate and chemical hazard (organism level).

The potential effects of climate change on the toxicity of chemical contaminants have received attention during the last two decades (Noyes et al., 2009). A promising research method for quantifying such effects are toxicokinetic models and experiments, which are used to assess how external concentrations (e.g., water concentrations) translate to internal concentrations (body burdens) through processes of uptake, elimination, biotransformation, and distribution (Gergs et al., 2019; Mangold-Döring et al., 2022). For most tested chemical–temperature interactions, there is an increase in toxicological sensitivity, but the interactions are species- and chemical-specific (Freitas et al., 2019; Huang et al., 2023). Temperature may also affect organism physiology (Polazzo et al., 2022) by influencing bioenergetics and fitness (Noyes & Lema, 2015). For an improved assessment of the combined effect of chemicals and temperature, it is important that the direct stress effect of temperature be incorporated into toxicokinetic-toxicodynamic (TK-TD) models so that an overall impact at the individual level is obtained. This in turn can be used in risk assessments or as an input for the assessment of population- or community-level effects (see the section “Climate and vulnerability to chemical stressors [community level]”).

Adverse outcome pathway (AOP) networks are another widely used approach, which facilitate describing and arraying evidence of potential chemical and nonchemical interactions from initiation events at the molecular level through to impacts on individuals (Hooper et al., 2013). They can be useful in providing evidence-based hypotheses and retrospective descriptions of how exposures to climate and chemical exposures may affect adverse outcomes. The Great Barrier Reef case study (Mentzel et al., 2023) exemplifies how AOP constructs can be developed to inform ERA, and how this can be aligned with probabilistic network models, and further developed into a quantitative model. However, most AOP networks developed to date do not account for toxicokinetic aspects, and are most applicable at the organism or sub-organismal level. Relevant data are often lacking to elaborate on biological pathways beyond the individual, resulting in a large uncertainty in the conceptual model as to multistressor effects on populations, and how these interact with ecosystems over time and larger landscapes (Rohr et al., 2016).

#### Climate and vulnerability to chemical stressors (community level).

A continuing challenge is understanding the combined effects of multiple stressors and at biological endpoints beyond the individual scale, in the context of ecological interactions and other environmental processes (Moe et al., 2013; Polazzo et al., 2022). This challenge is addressed by two of the case studies, where climate-related impacts on the vulnerability of individuals are extended to the population level (salmon populations) (Landis et al., 2023) and community level (coral reef) (Mentzel et al., 2023). Several promising modeling approaches are being developed for evaluating chemical and climate interactions at the community level (Bracewell et al., 2019; Turschwell et al., 2022). However, for decision-making purposes, there are still few evaluations of impacts to populations, communities, and habitats (Rohr et al., 2016), typically due to a lack of data. Understanding the spatial and temporal factors of these interactions is another important consideration at population and community levels. Conceptual models analogous to AOP networks but structured at higher biological and spatiotemporal scales may help identify the most critical climate-related processes that make biological assessment endpoints vulnerable to chemical stress, and thereby guide the development of relevant climate information for ERA.

The literature on the interaction between temperature (increased continuously or episodically) and contaminants at different levels of biological organization was recently reviewed by Bracewell et al. (2019) and Polazzo et al. (2022). Focusing on extreme climatic events, the latter paper identified only a few studies (13) that included biological effects of heat waves in combination with chemical pollution, and found that the reported combined effects varied largely with trophic level and with the type of endpoint (e.g., individuals or populations). As the interactive effects of chemical and climatic stressors can also be highly chemical-specific, it would be useful to group chemicals (and other stressors) by their mode of action (van den Brink et al., 2016). A refinement of this approach is urgently needed as it is impossible to evaluate the interactive effects of temperature for every chemical separately, so generalizations need to be made (van den Brink et al., 2019).

### Climate information: Robust statistical properties of climate projections

The term “climate information” (Nilsen et al., 2022) was introduced to the workshop by climate scientists and recommended for use as a bridge between climate models and impact studies. The workshop’s efforts to incorporate currently available climate information into the case study models, or to prepare the models for such developments in future projects, are summarized in Table 3. The term “climate model projections” is used to describe simulations of climate variables for future decades, produced by GCMs based on plausible scenarios for the concentrations of greenhouse gases and other relevant atmospheric constituents (see Table 4). Climatic impact drivers of importance to ERAs of chemicals include precipitation, droughts, and air temperature (Noyes et al., 2009). The intensity, frequency, and spatial extent of chemical risk often involve local and short-term events. Global climate models, in contrast, are only good at representing long temporal scales (e.g., decades) and larger regional scales (e.g., continents) (Benestad et al., 2023; Ranasinghe et al., 2021). For local applications to ERA, for example, to a river stretch, an understanding of climate impacts at a catchment scale is needed, which GCMs are not designed to provide. However, local climates depend on surrounding regional conditions and remote situations through teleconnections, such as the El Niño Southern Oscillation (Diaz et al., 2001; Doblas-Reyes et al., 2021). Therefore, local climate can be better approached by spatial downscaling of GCM projections.

Two main approaches for regional downscaling are commonly referred to as “top-down” and “bottom-up” (Pulido-Velazquez et al., 2022). The “top-down” approach involves downscaling climate projections from GCMs under a range of emission scenarios to provide inputs, for example, for models that predict impacts and analyze adaptation measures. Two common top-down ways to simulate regional and local climate conditions are “dynamical downscaling” through regional climate models (RCMs) and “empirical-statistical downscaling” through statistical modeling (Murphy, 1999). Both types of downscaling can involve various techniques with different assumptions, strengths, and weaknesses (Doblas-Reyes et al., 2021) and should, therefore be used in combination to make the most robust projections for the future. The output from RCMs (i.e., grid box area average) represents a larger spatial scale than historical observations (point measurements) and may require bias correction to be comparable to actual observations. Empirical–statistical downscaling, on the other hand, aims at reproducing similar aspects as those measured, and can also involve stochastic weather models known as weather generators. An alternative or supplement to the top-down approach is a bottom-up approach, where vulnerability thresholds and local responses are empirically studied to define locally suitable adaptation strategies (Pulido-Velazquez et al., 2022). Bottom-up approaches can make use of local knowledge through participative approaches to foresight future climate scenarios and define locally relevant adaptation strategies. Two examples of bottom-up methods for identifying what aspects are most important on a local scale are stress testing and sensitivity tests (Benestad, Parding, Mezghani, et al., 2019; Mtongori et al., 2015).

A key challenge is how to generate and make use of the available climate information in a way that can be accommodated by the ERA (Figures 1 and 2). The uncertainty related to several sources must be recognized (Beven, 2016; Kundzewicz et al., 2018), including choices of GCM models and downscaling techniques. The level of uncertainty is furthermore dependent on spatial and temporal scales of interest (see examples in Stahl Jr. et al. [2023]). A large set (ensemble) of GCMs can produce a plausible range of possible outlooks (Deser et al., 2012). However, a practical challenge for ERA is how to summarize the information provided by an ensemble in a way that captures the relevant uncertainties connected to regional climate variability. Benestad et al. (2023) suggested that the output from such ensembles often appears to follow a normal distribution, implying that the most relevant climate information can be captured by the mean and standard deviation, alternatively by other statistical types of distributions.

Aggregated information and statistical properties are often more predictable and more robust than individual outcomes due to “the law of small numbers,” making probabilistic representation of modeling results generally preferable to point estimates (Erlandsen et al., 2020; Moe et al., 2022). In addition, it is important that data and other information are relevant for the particular purpose and evaluated in a proper way, using methods of comparable complexity to appropriately capture the inherent uncertainty in both environmental changes and ecological responses (John et al., 2021). The production of climate information for a given ERA case (Figure 2B) should therefore be guided by the needs specified in the problem formulation, the selection of ecological assessment endpoints, and the identification of the main cause–effect relationships.

### Integration of climate and risk components: probabilistic methods

Bayesian networks are a type of probabilistic modeling method that has gained popularity for use in ERA (Kaikkonen et al., 2021) and that lends itself to the incorporation of probabilistic climate information. Bayesian networks are graphical models where the causal or empirical relationships between components (nodes) in the system are expressed by directional connections (arcs). The nodes are often defined by discrete states (e.g., intervals or categories), which are given probabilities that are conditional on their parent node values (Kjærulff & Madsen, 2013). Some examples of BNs in ERA are aligned with traditional frameworks (Figure 1B) and applied to calculate a risk quotient as a probability distribution derived from the ratio between exposure and effect distributions (Carriger & Barron, 2020; Mentzel, Grung, Tollefsen, et al., 2022). The BN Relative Risk Model (BN-RRM) (Landis, 2021) is a more advanced application based on a more strictly causal structure. The RRM principles emphasize the importance of multiple stressors and ecological assessment endpoints and the spatial overlap of endpoints’ habitats and occurrence of stressors.

While BN models are more commonly being used in ERAs of chemicals and other stressors, there are still few examples that involve climate model projections, to our knowledge (Gaasland-Tatro, 2016; Martínez-Megías et al., 2023; Mentzel, Grung, Holten, et al., 2022). In other fields, such as geosciences and hydrology, environmental assessments with BN models more often include climate models (e.g., Adams et al., 2022; Couture et al., 2018). However, these models often aim to predict contaminant concentrations rather than effects on biological endpoints.

Bayesian networks (or similar probabilistic network modeling methodologies) have several important features that are useful within the context of incorporating climate change into ERA. First, they allow for uncertainty quantification of different model components to be propagated throughout the network. Second, they are amenable to exploration of alternative scenarios, including scenarios of environmental management (e.g., pesticide application). Third, they allow quantification of environmental risk by both probability and consequence, in accordance with IPCC as well as other common definitions of risk. Fourth, calculations of relative risk applied to multiple ecological endpoints can be compared and ranked for different scenarios. Finally, they are particularly applicable for incorporating stakeholder knowledge and perspectives including uncertainty.

With this methodology, climate information in the form of probability distributions (Figure 1A) can be linked to one or more components of the risk characterization (Figure 1B) using conditional probability tables or other mathematical expressions that can propagate the uncertainty of the climate information. Existing examples of BNs that incorporate climate projections have typically used projections from only one or a few climate models, rather than from an ensemble as recommended recently (Moe et al., 2022). This is also the case with the case studies described in this paper (Table 1). However, the probabilistic structure of these models means that they are able to incorporate more robust climate information derived from model ensembles.

The flexible structure of BN models also allows for expanding the basic risk characterization to include climate-related vulnerability of ecological endpoints as a third risk component (Figure 2). This tripartite risk definition is inspired by the IPCC’s risk concept (Reisinger et al., 2020) and can allow more efficient use of information on climate effects on ecological components and interactions in assessments. In principle, this structure allows for explicit modeling of the concepts labeled climate-induced toxicant sensitivity and toxicant-induced climate sensitivity (Hooper et al., 2013; Moe et al., 2013). These concepts have also been used more recently in the context of temperature fluctuations and extreme events (Polazzo et al., 2022; Verheyen et al., 2019), although it might be difficult to distinguish the two phenomena in practice. Finally, the conditional probability tables of BN models can easily represent nonlinear relationships and statistical interactions between stressors, both of which are common in ecosystems. Shortcomings of the methodology are discussed in the section “Strengths and weaknesses of the proposed modeling approach.”

## CASE STUDIES

Three systems were selected as case studies to test this ERA modeling approach (Table 1), as described in the introductory paper (Stahl Jr. et al., 2023). These case studies were subsequently evaluated according to the three pillars of this modeling approach and the principles of incorporation of climate information as outlined earlier (Tables 2 and 3). The selected case studies (Table 1) represent three types of ecosystems: generic streams in agricultural areas in Norway (Oldenkamp et al., 2023), near-shore coral reefs in Australia (Mentzel et al., 2023), and a river network with salmonid populations in the United States (Landis et al., 2023). The three case studies were selected prior to the workshop to focus the discussions and to help develop and evaluate the risk modeling approach. While these case studies focus on a chemical stressor such as pesticides as well as nutrients, the approach presented here is meant to be generic and applicable for any type of chemical stressor.

Case study 1 was used in the European Union Innovative Training Network ECORISK2050 to explore new approaches for integrating climate projections with risk calculation, via the pesticide exposure model WISPE (Mentzel, Grung, Holten, et al., 2022). The climate projections that were applicable for this project resulted from the old climate scenario A1B and can now be considered obsolete but served the purpose of model development. Expected changes for northern Europe, such as increased rainfall, might result in increased pest pressure and pesticide emissions but could also be counteracted by improved agricultural practices or technologies (Hader et al., 2022). The WISPE tool was run by manual initiation with a climate file from individual climate models and does not readily allow efficiency for climate input from multimodel ensembles. However, by leveraging the existing input and output from WISPE based on two climate models (Mentzel, Grung, Holten, et al., 2022), the authors quantified key functional relationships between pesticide application rates, climatic variables (a monthly precipitation index), and peak exposures emerging from the WISPE model for individual pesticides. This functional relationship (regression models with associated uncertainty) can in turn be used to incorporate more updated climate information from ensemble models and to improve the pesticide exposure and risk assessment.

For Case study 2 (Mentzel et al., 2023), a BN model for near-shore coral reefs in the Mackay region of the Great Barrier Reef was built de novo based on a combination of extensive local data sources, the eReefs modeling database (CSIRO, 2015), regional climate projections, hydrological modeling, literature reviews, and expert judgment. The aim was to assess the collective risks of climate and catchment-related stressors on multiple endpoints for corals. An AOP network was used to conceptually delineate the effects of climate-related variables and the herbicide diuron on coral bleaching, mortality, and extent of cover. It illustrated both diuron-induced climate sensitivities and climate-induced diuron sensitivities (equivalent to “vulnerability” in Figure 2), which informed the conceptualization and development of the BN. The BN was used to quantitatively compare the effects of historic and future projected climate on inshore hard corals. It demonstrated how risk may be predicted for multiple physical and biological stressors including temperature, ocean acidification, cyclones, sediments, macroalgae competition, crown-of-thorns starfish (*Acanthaster planci*) predation, and chemical stressors such as nitrogen and herbicide exposure, provided that sufficient data and knowledge are available for model parameterization. Climate scenarios included an ensemble of 16 downscaled models encompassing current and future conditions based on two more recent greenhouse gas emission scenarios (RCP4.5 and 8.5).

Case study 3 was built upon a completed ERA model (Mitchell et al., 2021) to include climate scenarios and their impacts on Chinook salmon (*Oncorhynchus tshawytscha*) populations via water quality and pesticides. Key water quality input variables were adjusted based on a study of dissolved oxygen and temperature relationships across streams in the Sierra Nevada, California (Ficklin et al., 2013). Output from a 16-member ensemble of GCMs forced with the A2 emission scenario was statistically downscaled and used as input to the Soil Water and Assessment model at a subbasin level (Ficklin et al., 2013). As for Case study 1, climate projections that were already available for the region were used for model development, although resulting from an old climate scenario (A2). Future scenarios of pesticide concentration distributions were derived from in situ measured concentrations, by additional applications due to climate shifts as described by Stöckle et al. (2010), and decreased applications.

The three case study models were subjected to sensitivity analysis involving a quantification of the degree of mutual information between a target node (assessment endpoint) and the parent nodes, including both exposure- and climate-related variables. Outcomes from this sensitivity analysis varied across the case studies. For Case study 1, the hypothetical pesticide application scenarios had a stronger influence on the assessment endpoints than the main climatic variable (precipitation index). In contrast, in Case studies 2 and 3, the climate-related environmental variables had a stronger influence on the assessment endpoint than the chemical stressors (pesticides and nutrients). These outcomes suggest that when more ecologically relevant components are included in a risk assessment, such as the increased vulnerability of certain species or demographic stages at higher water temperatures, then climate-related factors can play an important role in the assessment relative to the chemical stressors.

The case studies were evaluated against each of the principles presented in Table 2. The evaluation demonstrated that the three study systems were all useful as case studies for exploring the proposed climate information and ERA modeling approach, but also that they all have potential for improvement. For example, all case study models contain conditional probability tables that include nonlinear cause–effect relationships as well as uncertainty. However, Case study 1 does not yet include cause–effect connections from exposure to effect (but instead an exposure*/*effect ratio), and Case study 2 has not yet integrated the various assessment endpoints representing the coral communities. While the modeling approach presented here builds upon the seven principles listed above, it has more explicit recommendations regarding the quantity, quality, and processing of climate model projections for integration with the risk components (as described in the section “Components of chemical risk and influence of climate change”). These aspects are listed in Table 3, which also presents an overview of how each of these pillars has been implemented in the case study models so far.

## DISCUSSION

### Strengths and weaknesses of the proposed modeling approach

The proposed modeling approach (Figure 2) aims to bridge a gap in scientific methodology at the intersection between climate modeling and environmental risk modeling, in particular, the handling of environmental variability and uncertainty. There are, as yet, few studies that combine the use of GCM ensembles with environmental impact analysis including ecological endpoints. Most assessment studies use climate projections from only one or a few GCMs, which implies a risk of bias due to low “sample size.” On the other hand, a large ensemble of GCMs implies methodological challenges in handling the vast array of climate projections. The approach described here can be further developed into a framework of statistical and probabilistic modeling methods to generate climate information, in a format that is both representative of key statistical properties of the climate model ensemble and relevant to the ecosystem of interest. However, successful integration of approaches from the two modeling fields will require close collaboration between climate experts and environmental assessment experts.

The environmental risk modeling described in this article and the case studies build upon the traditional ERA components of exposure and hazard characterization (Figure 1B) (Society of Environmental Toxicology and Chemistry, 2018), but also expand this to include an ecological vulnerability component, inspired by IPCC’s risk concept (IPCC, 2022; Reisinger et al., 2020). Moreover, all components and relationships can be quantified by probability distributions, as a more informative and flexible alternative than the single-value scores often used in ERA. The approach and BN implementation described here can also be adapted to other conceptual frameworks for environmental assessment and management, such as the Drivers–Pressures–States–Impacts–Responses (DPSIR) causal framework used by the European Environment Agency. Examples related to ecological assessment and management of water quality under climate change according to the EU Water Framework Directive are given by Moe et al. (2019) and see Cains et al. (2023), respectively.

The successful use of BN modeling for the purpose of ERA has increased during the last decade (Moe et al., 2021). Nevertheless, there are several methodological challenges associated with this method. Variables of BN models are normally (but not necessarily) discretized into states, which reduces the resolution and precision of the models, and can result in low sensitivity of a target node, for example, to changes in climate scenarios. The quantification of probability distributions will require more information, alternatively expert judgment, than a so-called deterministic (single-value) risk characterization. On the other hand, statistical methods such as hierarchical Bayesian models can be used for capturing even more information on uncertainty (e.g., of parameters) than what is common practice for BN models. Moreover, BNs cannot include feedback loops, although adaptations can be made. The BN models proposed here can be further advanced in several directions, for example, dynamic BNs for future projections allowing feedback loops (Gaasland-Tatro, 2016), spatially explicit BN models with links to geographical information systems (Piffady et al., 2021), cumulative risk of multiple stressors calculated as the joint probability of threshold exceedances, adapted to the specific type of stressor interaction (Welch, 2023), and decision-support tools with decision nodes and cost*/*benefit nodes (influence diagrams) (Rachid et al., 2021).

In local contexts, the uncertainties associated with global and downscaled climate projections may still be too large to be of practical use for decision-making. Therefore, alternative approaches such as physical climate storylines can be supplementary tools for incorporating value judgments and responses to policy options (Kunimitsu et al., 2023).

## CONCLUSION AND OUTLOOK

The novelty of this modeling approach lies in the production and use of robust climate information as a means to bridge multimodel ensemble climate modeling and probabilistic environmental risk modeling. The risk modeling can build upon the traditional ERA components of exposure and hazard characterization but may also include an ecological vulnerability component. Furthermore, a BN (or other probabilistic modeling methodologies) will allow all components and relationships to be quantified by probability distributions, as a more informative and flexible alternative than the single-value risk scores and assessment factors traditionally used in ERA.

This article has summarized and evaluated three relatively simple case studies from the SETAC Pellston workshop on global climate change and ERA (Stahl Jr. et al., 2023), each illustrating one or more aspects of the proposed modeling approach implemented as BN models. The application of our approach to three case studies has demonstrated the generality of the methodology across ecosystems and geographic regions. A more thorough model development and analysis of the case studies, which was beyond the scope of this workshop, should ideally include both climate projections from larger climate model ensembles, more recent climate scenarios, and both dynamical and empirical–statistical downscaling applied to all cases. More elaborate examples would also allow a more systematic evaluation of the costs and benefits of our proposed approach compared to alternatives.

We envision that the probabilistic modeling approach can help to meet the needs for improved methodology identified by scientific communities within both climate science and environmental toxicology science (Stahl Jr et al., 2017), as well as for other types of scientific assessments. The role of probabilistic modeling as a prerequisite for such integration must be recognized by scientists in both fields. The benefits of probabilistic risk assessment for an uncertain climate also need to be recognized by chemical risk managers (Cains et al., 2023) and other decision-makers whose decisions this framework is meant to support. As stated recently by climate scientists (Sillmann et al., 2018; Zscheischler et al., 2018), impact modelers and decision-makers need to work closely together with climate scientists to understand the complex events related to climate change.

## Figures and Tables

**TABLE 1:** Overview of the three case studies used to evaluate the proposed modeling approach to environmental risk assessment incorporating climate model projections. More details are given in the section case studies

Case study properties	Case study no. 1	Case study no. 2	Case study no. 3
Location and geographic region	Skuterud, Viken, Southeast Norway	Great Barrier Reef, Northeast Australia	Yakima River, Cascadia, Washington, Northwest USA
Ecosystem type	Stream	Near-shore coast	River
Chemical stressors and other stressors	Pesticides (trifloxystrobin, clopyralid)	Herbicides (diuron), nutrients (total nitrogen), sediments; biological (predation, competition)	Pesticides (malathion, diazinon, chlorpyrifos), dissolved oxygen
Risk assessment endpoint	Risk quotient (based on the ratio of PEC to NOEC or EC50)	Hard corals’ demographic rates and indicators (mortality, bleaching, cover)	Chinook salmon demographic rates and population size
Expected climate impacts on risk components (examples)	Precipitation—pesticide application and run-off (exposure); temperature—chemical degradation (exposure)	Precipitation—nutrient run-off (exposure); temperatur—coral bleaching (vulnerability); cyclones—physical damage (vulnerability)	Temperature and DO—fish development and survival (vulnerability)
Reference	(Oldenkamp et al., 2023)	(Mentzel et al., 2023)	(Landis et al., 2023)

Abbreviations: DO, dissolved oxygen; EC50, half-maximal effect concentration; NOEC, no observed effect concentration; PEC, predicted environmental concentration.

**TABLE 2:** Evaluation of the three case studies (Table 1) according to the seven principles of considering climate change within environmental risk assessment (Landis et al., 2013)

No.	Keywords	Case study 1: Agricultural stream (Norway)	Case study 2: Coral reef (Australia)	Case study 3: River salmon population (United States)
1	Importance of climate change (examples)	Higher temperatures and humidity are expected to increase the use of pesticides (especially fungicides).	Higher temperatures are expected to increase coral bleaching and vulnerability to other stressors.	Higher temperatures are expected to reduce dissolved oxygen and increase salmon vulnerability to other stressors. Climate change may change pest pressures and thus pesticide usage.
2	Assessment endpoints—ecosystem services	No: the endpoint is based on the traditional risk quotient (RQ).	Partly: hard corals of the Great Barrier Reef are associated with ecosystem services (e.g., aesthetic values, provision of habitat for other species, tourism), but not done explicitly here.	Yes: salmon population size is explicitly expressed as an ecosystem service (food provision, important part of culture of Indigenous communities).
3	Valuation of the endpoints	Higher risk to the stream community is measured by higher probability of exceeding a given RQ threshold.	Higher risk to the coral communities is measured by higher mortality, higher bleaching, and lower coverage.	Higher risk to the salmon population is measured by lower population size.
4	Multiple stressors; nonlinear effects	The current model is developed for pesticides as a single stressor, but can be expanded to represent the cumulative risk of multiple pesticides.	The model includes a chemical stressor (pesticide), other contaminant stressors (nutrients, sediments), biological stressors (predators, macroalgae), and climate-related stressors (heat, acidification, cyclones).	The model includes chemical stressors (pesticides) and climate-related stressors (temperature, hypoxia), but does not consider synergistic or antagonistic interactions.
5	Cause-effect diagrams	Partly: causal connections from climate and other scenarios to exposure, but not from exposure to effect.	Partly: causal connections from climate scenarios and other scenarios to several coral endpoints, but the endpoints are not integrated.	Yes: all connections from climate and other scenarios represent cause–effect and are integrated into one endpoint (population size).
6	Uncertainty and sensitivity	Sensitivity analysis showed high influence of pesticide emission scenarios relative to climate.	Sensitivity analysis showed high influence of climate (cyclones, heat) relative to catchment-related stressors (nutrients and pesticides).	Sensitivity analysis showed higher influence of water quality-related variables than of pesticide application scenarios.
7	Adaptive management	Not considered explicitly; management relevance is considered at higher policy level (e.g., the EU Green Deal).	Considered explicitly, with an emphasis on improving water quality to enhance reef resilience to climate stressors.	Considered explicitly: for example, the importance of habitat protection and management changes in different seasons.

**TABLE 3:** Mapping of the case studies (Table 1) according to the three pillars of the proposed modeling approach for integrating climate model projections with environmental risk assessment

Pillar no.	Keywords	Case study 1: Norway	Case study 2: Australia	Case study 3: United States
(1) Climate information: preparation of climate model projections for case study	Recent IPCC scenarios	No (A1B)	Yes (RCP4.5, RCP8.5)	No (A2)
	Multiple GCMs (ensemble)	No (2 GCMs)	Partly: four GCMs (selected for their efficacy from a detailed assessment of all models included in the World Climate Research Programme’s Coupled Model Inter-comparison Project)	16 GCMs used in Ficklin et al., 2013 to derive DO and temperature adjustments
	Regional downscaling: empirical–statistical and/or dynamic	Empirical (1 RCM)	Three statistical downscaling methods and one dynamic downscaling method used	Statistical downscaling used in Ficklin et al., 2013 to derive DO and temperature adjustments
	Processing of climate projections as relevant information	Yes: monthly precipitation index from daily values	Daily and monthly	Daily precipitation, temperature, and wind speed rescaled to monthly means (Ficklin et al., 2013)
	Time horizon	Present, 2050, 2085	Present, 2040, 2085	Present, 2050, 2080
(2) Quantification of climate effect on all risk components	Exposure, directly affected by climate (environmental)	Yes: functional relationship precipitation—pesticide runoff derived from a process-based model	Partly: Functional relationship between rainfall, total nutrient load, and sediments; air temperature, sea temperature. No relationship between runoff and diuron	Yes: Temperature and dissolved oxygen predicted by a catchment model (SWAT)
	Exposure, indirectly affected by climate (anthropogenic)	Partly: plausible pesticide application scenarios	Yes	Yes
	Hazard (toxicity or sensitivity to exposure in lab)	No	No	Yes
	Vulnerability (to exposure in field)	No	Yes: for example, mortality due to temperature and cyclones	Yes: reduced egg-to-emergence and adult survival
(3) Use of a probabilistic method to represent uncertainty in climate information, risk components, and their connections	Uncertainty in climate information	Yes: temporal variability in precipitation (between-years)	Yes	Yes
	Uncertainty in chemical exposure	Yes: including predicted between-year variation	Yes: resulting from conditional probability tables (CPTs)	Yes: from distributions of monitoring data
	Uncertainty in chemical effect (hazard and/or vulnerability)	Yes: from interspecies variation in NOEC or EC50	Yes: resulting from CPTs	Yes: from multiple population model runs
	Uncertainty in links from climate to risk components	Exposure—yes: from estimated uncertainty in functional relationship	Effects—partly: in some CPTs from expert judgment or from equations	Yes: temperature and DO were adjusted with distributions from ensemble modeling outputs. Pesticides adjusted with simple percentage reductions

Abbreviations: CPTs, conditional probability tables; DO, dissolved oxygen; GCM, global climate model; IPCC, Intergovernmental Panel on Climate Change; NOEC, no observed effect concentration; RCM, regional climate model; SWAT, Soil and Water Assessment Tool.

**TABLE 4:** Explanation of important terms and abbreviations used in this article

Term	Explanation
Climate change	Long-term shifts in temperatures and weather patterns, primarily due to burning fossil fuels like coal, oil, and gas. (*Source*: https://www.un.org/en/climatechange/what-is-climate-change).
Climate information	Quantitative information derived from climate model projections and represented by statistical properties, aimed to be robust to model assumptions, representative of the most recent knowledge available, and relevant for the study area.
CMIP	Climate Model Intercomparison Project: A collaborative framework designed to improve knowledge of climate change organized by the WGCM of the WCRP to foster the climate model improvements and to support national and international assessments of climate change.
CORDEX	Coordinated Regional Climate Downscaling Experiment; an international framework coordinating international work on downscaling under the WCRP.
Downscaling	A process that makes it possible to estimate the response in local temperature or precipitation to global warming. The two main methods are (1) dynamical downscaling with RCMs and (2) (empirical–)statistical downscaling. Downscaling can also introduce errors and inaccuracies.
ERA	Ecological Risk Assessment (more commonly used in North America); Environmental Risk Assessment (more commonly used in Europe; can be used in North America to include ecological + human health risk assessment).
GCM	Global Climate Models: Models simulating earth’s atmosphere, and ocean and land processes. Also referred to as General Circulation Models.
Model predictions	For atmospheric and oceanic models, predictions are those simulations that use the best description that we have of the current state of the atmosphere and calculate the most likely development some time ahead. This includes seasonal and decadal forecasts. We say that these models are “initialized.”
Model projections	Simulations with climate models constrained by a given future development in external factors (“boundary conditions”), such as greenhouse gases or land-use changes. Model projections do not necessarily start with a best description of the current state, but with a plausible scenario. The CMIP simulations are typically referred to as “climate change projections.”
RCM	Regional Climate Model: A climate model forced by specified lateral and ocean conditions from a GCM.
RCP	Representative Concentration Pathways: Scenarios of greenhouse gas concentrations and radiative forcing used for climate modeling.
SSP	Shared Socioeconomic Pathway: Scenarios of projected socioeconomic global changes up to 2100.
WCRP	World Climate Research Programme: An international program to coordinate global climate research, established in 1980 under the joint sponsorship of the World Meteorological Organization (WMO) and the International Council for Science (ICSU), and has also been sponsored by the Intergovernmental Oceanographic Commission (IOC) of UNESCO since 1993.
WGCM	Working Group on Coupled Modeling, with the overall mission to foster the development and review of coupled climate models.

## Data Availability

There are no data directly associated with this article. The data availability of the case studies is described in the individual case study papers cited herein.
